# Genetic Diversity of *Nocardia cyriacigeorgica* Isolated from Bovine Mastitis in Two Chinese Dairy Herds

**DOI:** 10.3390/ani15223229

**Published:** 2025-11-07

**Authors:** Deyuan Song, Yan Zhao, Hao Li, Meiyi Ren, Ziyan Wang, Qinna Xie, Mingchao Liu, Jian Gao, Jia Cheng

**Affiliations:** 1 Department of Clinical Veterinary Medicine, College of Veterinary Medicine, Hebei Agricultural University, Baoding 071001, China; s826718143@163.com (D.S.); zhaoyan200012@163.com (Y.Z.); 20242200643@pgs.hebau.edu.cn (H.L.); renmeiyi@pgs.hebau.edu.cn (M.R.); 20247201748@pgs.hebau.edu.cn (Z.W.); 15230928849@163.com (Q.X.); liumingchao@163.com (M.L.); 2 Department of Clinical Veterinary Medicine, College of Veterinary Medicine, China Agricultural University, Beijing 100193, China

**Keywords:** *Nocardia cyriacigeorgica*, bovine mastitis, environment, genetic diversity

## Abstract

**Simple Summary:**

Mastitis remains one of the most prevalent and economically important diseases in the dairy industry. Among the diverse pathogens associated with mastitis, *Nocardia cyriacigeorgica* has been increasingly recognized in several countries; however, its occurrence on farms and the extent of strain variation have not been well characterized. In the present study, two large Chinese dairy herds experiencing mastitis outbreaks were systematically investigated. Milk samples were collected from cows with clinical, subclinical, and healthy udders, alongside a wide range of environmental samples. *N. cyriacigeorgica* was detected in both milk and environmental sources, with the highest detection rates observed on teat skin, nasal swabs, and milking equipment. Analyses of genetic diversity showed that the isolates were highly similar, suggesting repeated exposure to contaminated environments. These findings provide new insight into the ecology of *N. cyriacigeorgica* in dairy herds and highlight the importance of environmental management and hygiene practices in reducing infection pressure and improving udder health.

**Abstract:**

Mastitis continues to impose considerable economic losses on the dairy industry; however, the epidemiological characteristics and genetic diversity of *Nocardia cyriacigeorgica* remain poorly characterized. In the present study, two large-scale Chinese dairy farms experiencing mastitis outbreaks were investigated, with a total of 666 milk and 288 environmental samples collected. From the milk samples, 85 isolates of *N. cyriacigeorgica* were identified, corresponding to detection rates of 22.7% in clinical mastitis, 11.2% in subclinical mastitis, and 28.0% in healthy milk. Whole genome sequencing combined with multilocus sequence analysis demonstrated markedly limited genetic diversity, with the majority of isolates clustering within a few closely related lineages (Simpson’s Index: 0.12 for clinical and 0.037 for subclinical samples). Furthermore, qPCR screening detected *N. cyriacigeorgica* in 21.9% of environmental samples, with the highest detection frequencies observed on nasal swabs (62.5%), teat skin (56.3%), interdigital spaces (56.3%), and milking equipment (45.8%). Taken together, these findings are consistent with shared environmental exposure within farms and suggest a role for persistent reservoirs in repeated cow exposure. This investigation provides a comprehensive characterization of the population structure and distribution of *N. cyriacigeorgica* in Chinese dairy herds and underscores the importance of environment-focused management strategies for reducing infection pressure and enhancing mastitis control.

## 1. Introduction

Bovine mastitis is a widespread and economically impactful disease that remains a persistent challenge due to the diverse and complex nature of its causative pathogens [1]. Its etiology involves a broad spectrum of microorganisms, including *Staphylococcus aureus*, non-aureus staphylococci, *Streptococcus* spp., *Escherichia coli*, and *Klebsiella* spp. [2]. *Nocardia* spp., a group of filamentous, branching Gram-positive bacteria, have long been recognized as causative agents of localized or systemic suppurative diseases in both humans and animals [3]. The predominant *Nocardia* species associated with bovine mastitis include *N. asteroides*, *N. farcinica*, *N. nova*, *N. otitidiscaviarum*, and *N. cyriacigeorgica* [4]. Notably, *Nocardia* spp. have also been identified as etiological agents of mastitis in several countries, including the United Kingdom [5], Italy [6], Brazil [7], and China [8], where infections frequently result in abscess formation, inflammation, and decreased milk production.

Widely distributed in soil, water, and both the internal and external environments of various animals and plants, *Nocardia* species are generally regarded as environmental pathogens [9,10]. Although capable of causing severe invasive diseases, *Nocardia* spp. are primarily opportunistic pathogens in both humans and dairy cattle. Notably, the incidence and clinical burden of human nocardiosis vary significantly across different geographical regions [11].

The application of molecular epidemiology has greatly advanced our understanding of the population structure and diversity of pathogens associated with bovine mastitis [12,13,14]. Analyzing bacterial genetic diversity is essential for characterizing epidemiological patterns and distinguishing strain-level variations [15]. Various molecular typing methods have been employed to investigate the genetic diversity of *Nocardia* species, including 16S rRNA sequencing and multilocus sequence analysis (MLSA) [16,17]. Among these, MLSA has proven to be a highly discriminative technique for identifying clinical *Nocardia* isolates [18]. These molecular approaches have played a critical role in clarifying genetic relationships and supporting epidemiological studies of bacterial pathogens [11].

A comprehensive investigation of the microbiological agents and their pathogenic mechanisms underlying bovine mastitis is essential for developing effective prevention and control strategies. Although the pathogenicity of *Nocardia* species in humans and other animals has been documented, their presence, genetic diversity, and association with mastitis in dairy herds remain underexplored. In particular, the role of *Nocardia cyriacigeorgica* as a potential reservoir in extramammary and environmental niches on dairy farms has not been clearly established. Therefore, the objective of this study was to determine the phylogenetic characteristics of *N. cyriacigeorgica* isolated from cases of clinical mastitis (CM) and subclinical mastitis (SCM) on two large-scale dairy farms in China.

## 2. Materials and Methods

### 2.1. Farms

This study was conducted in May 2023 on two large-scale free-stall dairy farms (farm A and farm B) located in Hebei and Shandong Province, China, both of which had reported outbreaks of clinical mastitis (CM). Following initial contact from the farm management teams, researchers visited the farms to collect milk and environmental samples (Table 1). At the time of the outbreak, farm A housed 4770 lactating Holstein-Friesian cows, whereas farm B maintained a larger herd of 11,092 lactating cows. All cows were fed a total mixed ration (TMR) consisting of alfalfa, oat hay, corn silage, and concentrate supplements. Milking was performed three times daily, using a 2 × 32 parallel rapid-exit milking parlor at farm A and a 64-stall rotary milking parlor at farm B, with the milking machines maintained on a monthly basis. Cows were housed in 1000-cow freestalls arranged in groups of 250 animals, with connecting corridors leading to the milking area. Bedding material on both farms consisted of recycled manure solids (50–60% dry matter), produced via a screw-press separator system. Milking personnel were trained to recognize clinical signs of mastitis; however, awareness of subclinical mastitis (SCM) remained limited.

### 2.2. Nocardia Clinical, Subclinical Mastitis Cases

An outbreak of mastitis occurred on two dairy farms (farm A and farm B) in May 2023, characterized by a marked increase in clinical mastitis (CM) cases with severe symptoms. Prior to any antibiotic treatment, veterinarians collected milk samples aseptically from affected quarters of all cows presenting with CM, based on the presence of abnormal milk, udder swelling, or both. Subclinical mastitis (SCM) cases were identified using the California Mastitis Test (CMT), and corresponding quarter milk samples were similarly collected. At farm A, within the first 30 days following our visit, 53 *Nocardia* isolates were obtained from 431 SCM samples, and 1 isolate was obtained from 45 CM samples collected by farm veterinarians. At farm B, within 60 days of the visit, 1 *Nocardia* isolate was identified from 50 SCM samples, 16 isolates from 30 CM samples, and 14 isolates from 50 healthy milk samples (HEAL), which were collected from cows with no clinical or subclinical mastitis. These HEAL samples exhibited negative CMT results, indicating no inflammation. In addition to *Nocardia*, other bacterial pathogens were isolated from clinical mastitis (CM) samples. At farm A, *Streptococcus uberis* was detected in 6.09% of the samples, while coagulase-negative staphylococci (CNS) accounted for 21.9%. At farm B, *Escherichia coli* was found in 10.62% of CM samples, and *Streptococcus uberis* in 10.22%. All bacterial identification procedures were conducted in accordance with the guidelines established by the National Mastitis Council [19].

### 2.3. Milk Sample Collection and Molecular Identification

All milk samples from affected quarters of all cows with CM before treatment were collected by veterinarians on these two dairy farms. California Mastitis Test (CMT) (DeLaval, Tumba, Sweden) was used to identify quarters with SCM [20]. A total of 431 quarter milk samples were aseptically collected from non-repetitive SCM quarters among 4770 lactating cows at farm A, and 50 quarter milk samples were collected from 11,092 cows at farm B, within 3 to 5 days after the research team’s initial visit. In addition, 50 quarter milk samples were randomly collected from clinically healthy cows at farm B to serve as healthy controls. The health status of these cows was reconfirmed prior to grouping, based on negative CMT results, somatic cell counts (SCC) below 200,000 cells/mL, and the absence of *Nocardia* spp. growth upon culture. Bulk tank milk (BTM) samples were also obtained from both farms (*n* = 30 per farm) by researchers from Hebei Agricultural University (Baoding, Hebei Province, China) for comparative analysis.

A total of 53 quarter milk samples at farm A and 1 sample at farm B tested CMT-positive, as determined by the farm veterinarians at each location. Milk samples were subsequently cultured using bacteriological procedures described previously [21]. Briefly, 10 μL of each milk sample was inoculated onto a 5% bovine blood agar plate and incubated aerobically at 37 °C for 48 to 72 h. Samples were considered culture-positive if one or more colonies were observed, with a threshold of ≥100 colony-forming units per milliliter (CFU/mL) [22]. Samples yielding ≥3 distinct bacterial species were classified as contaminated, except when *Staphylococcus aureus* or *Streptococcus agalactiae* were present. Suspected *Nocardia* colonies were identified based on typical morphological characteristics on blood agar plates and further subjected to confirmatory biochemical tests, including Gram staining, indole production, ornithine decarboxylation, and oxidase activity. Final species-level confirmation was performed via 16S rDNA gene sequencing.

### 2.4. Antimicrobial Susceptibility Testing

The antimicrobial susceptibility of *N. cyriacigeorgica* isolates was assessed using the broth microdilution method for determination of minimum inhibitory concentrations (MICs), following the Clinical and Laboratory Standards Institute (CLSI) guidelines (M24S, 2023) [23]. Bacterial suspensions were prepared in Middlebrook 7H9 broth, adjusted to a turbidity equivalent to the 0.5 McFarland standard (≈1.5 × 10^8^ CFU/mL), and subsequently subjected to three sequential 10-fold dilutions to yield a final inoculum density of approximately 1.5 × 10^5^ CFU/mL. Two-fold serial dilutions of each antimicrobial agent were prepared in sterile 96-well microtiter plates containing 100 μL of 7H9 broth per well. Following inoculation with 100 μL of the standardized bacterial suspension, the plates were incubated at 37 °C for 72 h. Appropriate controls were included in each assay, with the 11th column serving as a positive growth control (medium with inoculum but no antimicrobial agent) and the 12th column serving as a negative control (medium only). *Nocardia nova* ATCC BAA-2227 was used as the quality control reference strain to ensure assay reliability.

### 2.5. Extramammary and Environmental Samples Collection and DNA Extraction

A total of 288 extramammary and environmental samples were collected, with 144 samples respectively collected from farms A and B under identical environmental sources. Cow level (teat skin, teat canal, interdigital spaces, nasal, saliva, cow hoof and feces) and the milking parlor level (milking equipment, hands of the milking personnel, milking parlor aisle) were used PBS (Solarbio, Beijing, China)-imbued sterile cotton swab, the cowshed level (freshly prepared clean bedding in the dairy farm, residue left after wet and dry separation of cow manure, bedding, soil under the bedding of the perinatal barn, feed, water) was stored in clean, ziplock bags, and within 1 h were diluted in 20 mL of sterile PBS (Table 2). All collected environmental samples were transported to the laboratory at Hebei Agricultural University and stored at −20 °C until use. For each environmental sample, 50 mg was taken and 2 mL of lysozyme (BioTeke, Beijing, China) was added. Swab samples were sheared and placed into sterile centrifuge tubes containing the lysate. All samples were ground with steel balls, and 2 mL of lysozyme was added again. Following the manufacturer’s instructions, genomic DNA was extracted from all lysozyme-treated environmental samples using a bacterial DNA extraction kit (BioTeke, Beijing, China). The integrity of the genomic DNA was tested by electrophoresis. The crude DNA samples were frozen at −20 °C for storage.

### 2.6. TaqMan qPCR

After aligning the *Nocardia* 16S rRNA gene sequences registered in NCBI using MEGA 7.0, a conserved sequence was selected. Based on this sequence, a pair of primers and a probe were designed using Primer Express 3.0 software. The fluorescent probe was labeled with a carboxy fluorescein (FAM) fluorescent group at the 5′ end and a Black Hole Quencher-1 (BHQ1) quenching group at the 3′ end (Table 3).

Using specific primers and probes, *Nocardia* was detected in 144 extramammary and environmental samples (teat skin, teat canal, interdigital spaces, nasal, saliva, cow hoof, feces, milking equipment, hands of the milking personnel, milking parlor aisle, freshly prepared clean bedding in the dairy farm, residue left after wet and dry separation of cow manure, bedding, soil under the bedding of the perinatal barn, feed, water). Ct (cycle threshold) values were recorded as the number of amplification cycles required for fluorescence to exceed the background threshold. Mean Ct values were calculated for each sample type, with lower Ct values corresponding to higher bacterial DNA concentrations.

### 2.7. Whole Genome Sequencing and Bioinformatic Analysis

A total of 85 *Nocardia* isolates isolated from the CM, SCM and HEAL cases of cows were submitted for whole genome sequencing. The FASTQ format row data sequence was obtained after sequencing. Spades software (3.15.4 version) was used to splice the raw data, and prodigal (v2.60) software was used to translate the spliced sequence into amino acid sequences. Then, Orthofinder (2.4.0 version) was used to search for single-copy orthologous genes, and phylogenetic trees were constructed using single-copy orthologous genes. After mafft (7.505 version) comparison and trimal pruning, the iq-tree evolutionary tree was established, and finally the Interactive Tree of Life (iTOL) online tool (version 6) was used for constructing a phylogenetic tree.

### 2.8. Molecular Typing of 16S rRNA Sequencing, MLSA

According to CLSI (2018) [24], strains showing ≥99.6% similarity in 16S rRNA sequences were typically considered conspecific. To perform phylogenetic comparisons, raw sequences were initially compiled using SEQ-Man (DNASTAR, Madison, WI, USA) and later standardized with BioEdit to match the length of the shortest reference (1482 bp). The aligned sequences were analyzed phylogenetically with the ClustalW algorithm, and phylogenetic trees were constructed using MEGA 7.0, employing both neighbor-joining (NJ) and maximum likelihood (ML) methods with 1000 bootstrap replicates. For multilocus sequence typing (MLST), seven housekeeping genes (gyrB, hsp65, secA1, rpoB, rpoA, recA, and trpB) were used to genotype *Nocardia cyriacigeorgica* isolates from milk samples. Currently, Multilocus sequence analysis (MLSA) is widely recognized as a reliable molecular approach for distinguishing various *Nocardia* taxa. By using 16S rDNA–*gyrB*–*hsp65* MLSA analysis, *Nocardia* can be accurately identified [25]. The multilocus sequence analysis (MLSA) based on five gene loci (16S rRNA, *gyrB*, *secA1*, *hsp65*, and *rpoB*) also plays an important role in the identification of *Nocardia* isolates [26]. The development of multilocus sequence typing (MLST) based on seven housekeeping genes enables the genotyping analysis of *Nocardia* species [16]. MLSA was performed using trimmed sequences of concatenated 16S rRNA-*gyrB*-*hsp65* (3168-bp), *gyrB*-16S rRNA-*secA1*-*hsp65*-*rpoB* (4151-bp) and *gyrb*-*hsp65*-*secA1*-*rpoB*-*rpoA*-*recA*-*trpB* (5938-bp). An NJ phylogenetic tree was then constructed using MEGA 7.0 [27,28].

### 2.9. Statistical Analyses

Data analysis of *Nocardia* strains isolated from CM, SCM, and HEAL cases at farms A and B was performed using R version 4.4.3 (https://cloud.r-project.org/), with a significance threshold set at *p* < 0.05. Data visualization was conducted using GraphPad Prism version 8.0. Samples were categorized into cowshed, milking parlor, and cow-related. A multivariate logistic regression model was applied using *N. cyriacigeorgica* detection (qPCR result) as the binary outcome, with cowshed samples as the reference group. Odds ratios (ORs) with 95% confidence intervals (CIs) were calculated, with significance set at *p* < 0.05. Analyses were conducted in R 4.4.3 using the glm() function and the broom package.

Gene sequences of *16S rRNA*, *gyrB*, *hsp65*, *secA1*, *rpoB*, *rpoA*, *recA*, and *trpB* were aligned using MEGA (v7.0) and concatenated. Unrooted phylogenetic trees were constructed using the neighbor-joining (NJ) method with the Kimura 2-parameter model. Bootstrap values (>70%) from 1000 replicates were shown at branch nodes. Clusters (I–III) were manually assigned based on clear phylogenetic separation and strong bootstrap support (≥70%). No automated clustering or fixed threshold was used.

The Simpson’s Index of Diversity (SID) and its corresponding 95% confidence interval (CI) were estimated according to farm and different sample types, using the following equation:

$${SID}_{S}=1-\sum \left(\frac{n_{i}\left(n_{i}-1\right)}{N\left(N-1\right)}\right)$$

where N is the total number of samples. n_i_ is the number of isolates assigned to the ith specific genotype.

## 3. Results

### 3.1. Isolation Rate of N. cyriacigeorgica

A total of 85 *N. cyriacigeorgica* isolates were detected in CM, SCM, and HEAL samples from the two dairy farms. At farm A, 1 (2.2%) isolate was recovered from CM samples and 53 (12.3%) isolates from SCM samples. At farm B, 16 (53.3%) isolates were recovered from CM samples and 1 (2.0%) isolate from SCM samples. Additionally, 14 (28.0%) *N. cyriacigeorgica* isolates were detected in HEAL samples from farm B. No *N. cyriacigeorgica* was isolated from BTM at either farm A or B (Table 4).

### 3.2. Antimicrobial Susceptibility Results

Antimicrobial susceptibility testing of the 85 *N. cyriacigeorgica* isolates was performed using the broth microdilution method in accordance with CLSI M24S (2023) guidelines [23]. The MIC distributions and resistance rates for seven commonly used antimicrobial agents are summarized in Table 5. All isolates exhibited complete resistance to clarithromycin and rifampin, while high resistance was also observed for ciprofloxacin (92.9%). In contrast, all isolates remained susceptible to amikacin and tobramycin. Intermediate levels of resistance were observed for moxifloxacin (28.2%) and doxycycline (31.8%).

### 3.3. Whole Genome Sequencing and Bioinformatics Analysis

**Figure 1:**
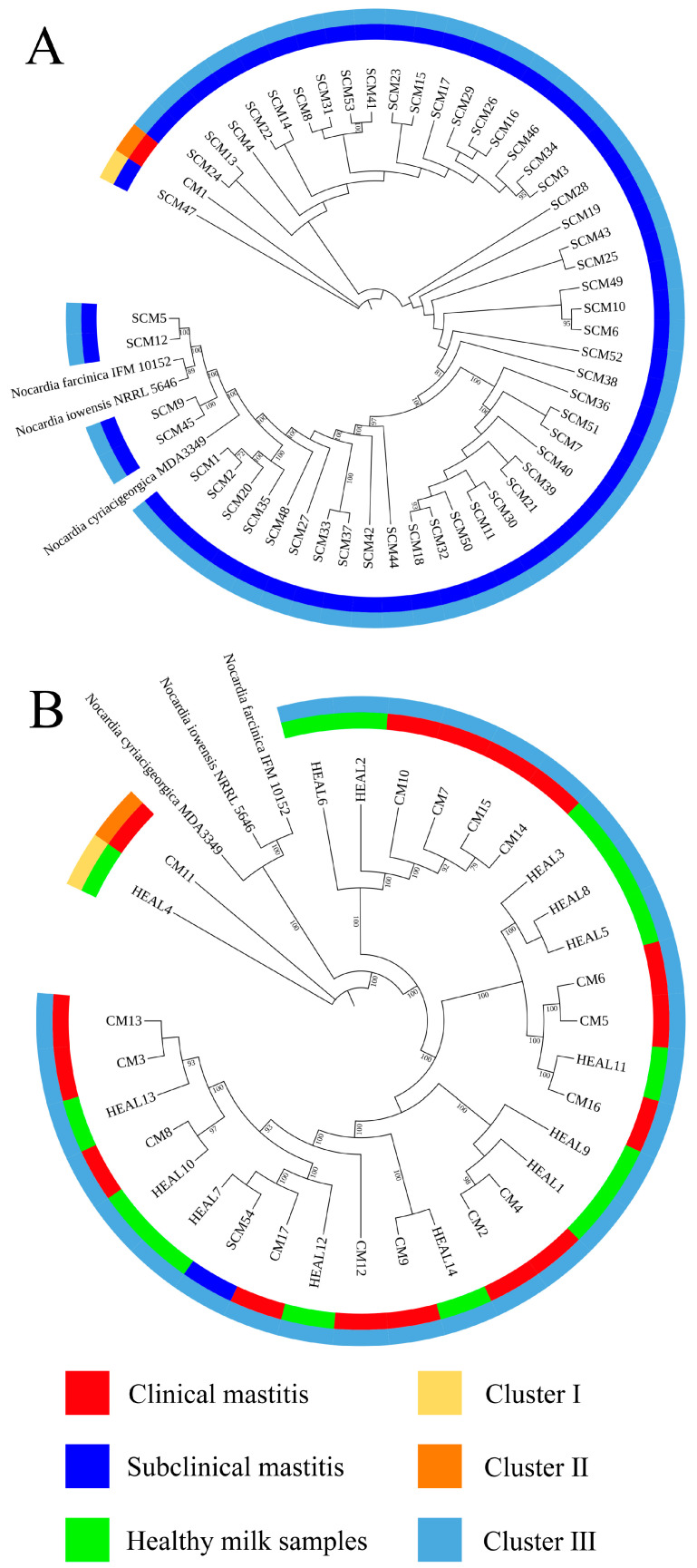
(**A**) Phylogeny tree of 54 *Nocardia cyriacigeorgica* using whole genome sequencing in farm A. (**B**) Phylogeny tree of 31 *Nocardia cyriacigeorgica* using whole genome sequencing in farm B. The scale bars represent 0.1 substitutions per nucleotide position.

Whole genome sequencing was performed on 54 *Nocardia cyriacigeorgica* isolates obtained from farm A. Among these, 1 isolate from CM was classified into cluster II. For the SCM isolates, 1 was grouped into cluster I and 52 into cluster III, resulting in an SID of 0.038 (95% CI: 0.029–0.049) (Figure 1A).

At farm B, phylogenetic analysis was conducted on 31 *N. cyriacigeorgica* isolates. 1 SCM isolate was classified into cluster III. Among the CM isolates, one was grouped into cluster II and fifteen into cluster III, yielding an SID of 0.13 (95% CI: 0.082–0.19). For HEAL samples, 1 isolate was assigned to cluster I and thirteen to cluster III, with an SID of 0.14 (95% CI: 0.092–0.22) (Figure 1B).

**Figure 2:**
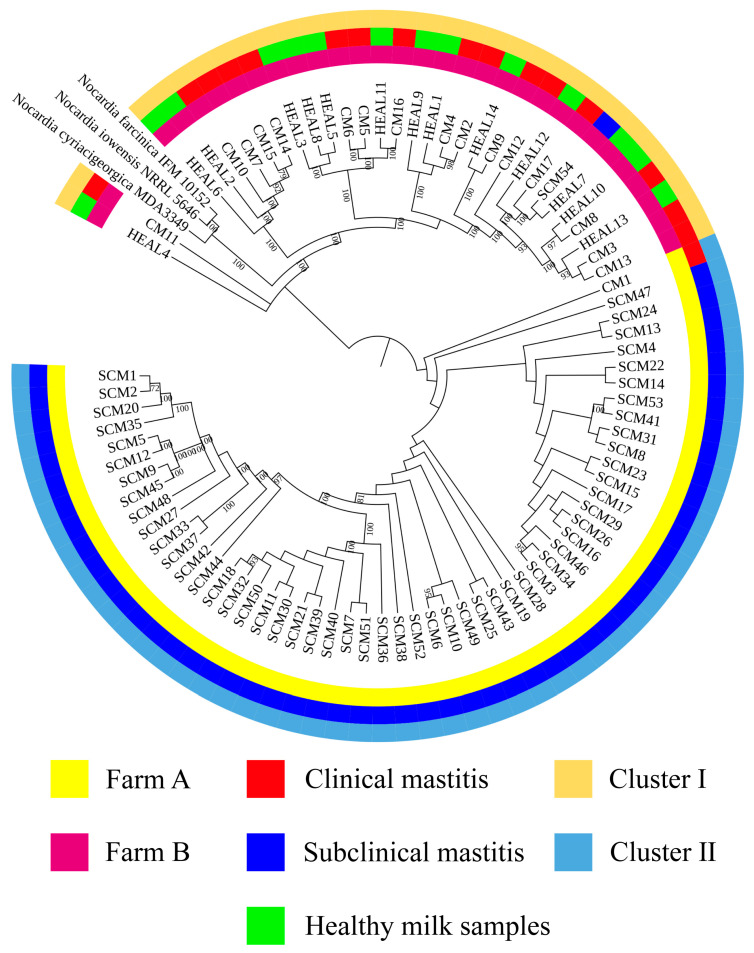
Phylogeny tree of 85 *Nocardia cyriacigeorgica* using whole genome sequencing in farms A and B. The scale bars represent 0.1 substitutions per nucleotide position.

In the combined analysis of all 85 isolates from farms A and B, 14 HEAL isolates were classified into cluster I. Among the CM isolates, 16 were grouped into cluster I and 1 into cluster II, with an SID of 0.12 (95% CI: 0.078–0.17). For SCM, 1 isolate was classified into cluster II and 53 into cluster I, resulting in an SID of 0.037 (95% CI: 0.029–0.048) (Figure 2).

### 3.4. Phylogenetic Analyses on MLSA

#### 3.4.1. 16S rRNA-*gyrB-hsp65*

**Figure 3:**
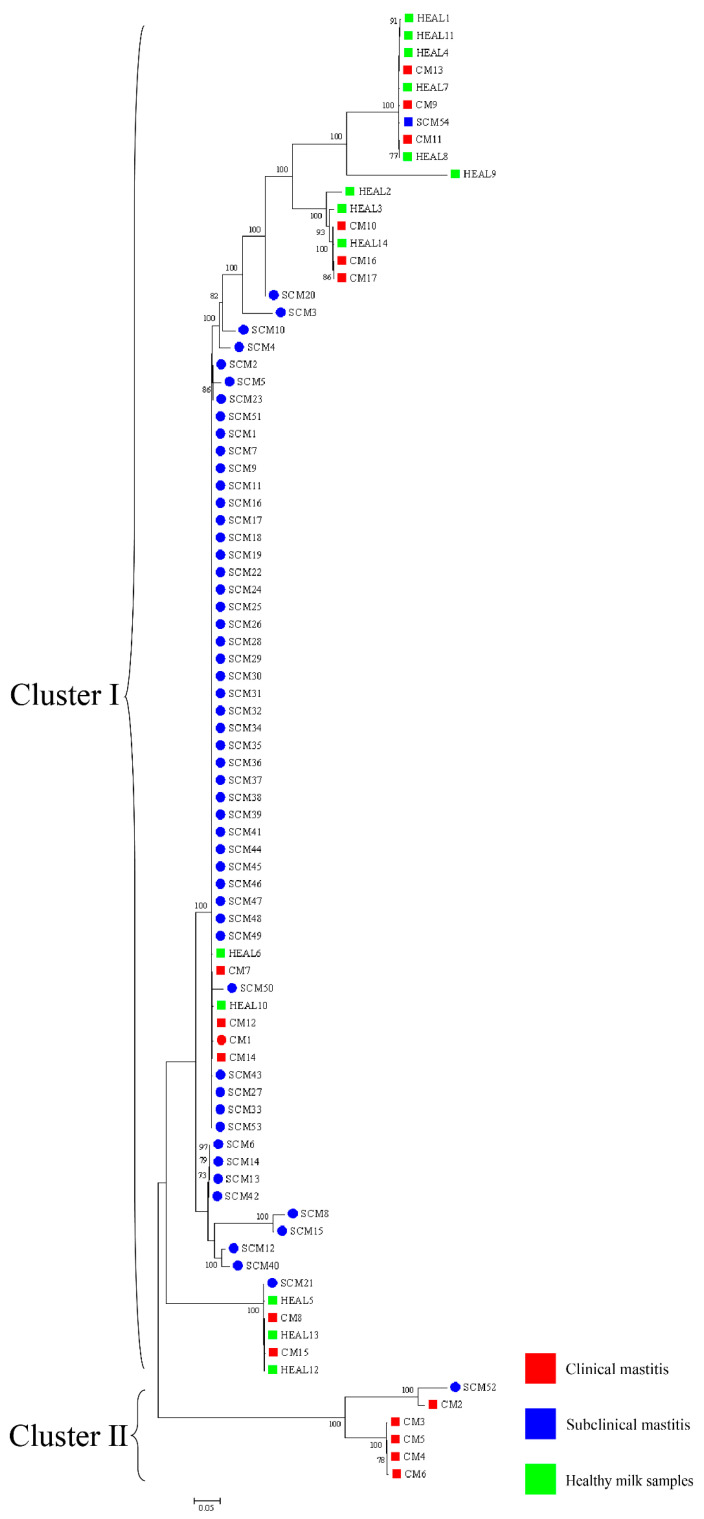
NJ trees based on the 16S rRNA-*gyrB*-*hsp65* gene sequences presenting the phylogenetic relationships of *Nocardia* isolated from clinical mastitis, subclinical mastitis, and healthy milk samples. The scale bars represent 0.05 substitutions per nucleotide position. ○ represents farm A, □ represents farm B.

Phylogenetic analysis was performed based on a combination of three genes (16S rRNA-*gyrB*-*hsp65*) containing all isolates (Figure 3). In the overall analysis of all 85 *Nocardia cyriacigeorgica* isolates, 14 isolates from HEAL samples were classified into cluster I. Among the CM isolates, 12 were grouped into cluster I and 5 into cluster II, yielding a Simpson’s Index of Diversity (SID) of 0.44 (95% CI: 0.37–0.52). For SCM, 53 isolates were assigned to cluster I and 1 to cluster II, with an SID of 0.037 (95% CI: 0.029–0.048). At farm A, 1 isolate from a CM case was classified into cluster I. Among SCM isolates, 52 were grouped into cluster I and 1 into cluster II, resulting in an SID of 0.038 (95% CI: 0.029–0.049). At farm B, 14 isolates from HEAL samples and 1 from SCM were all classified into cluster I. Among CM isolates from this farm, 11 were grouped into cluster I and 5 into cluster II, with an SID of 0.46 (95% CI: 0.38–0.54).

#### 3.4.2. *gyrB*-16S rRNA-*secA1*-*hsp65*-*rpoB*

**Figure 4:**
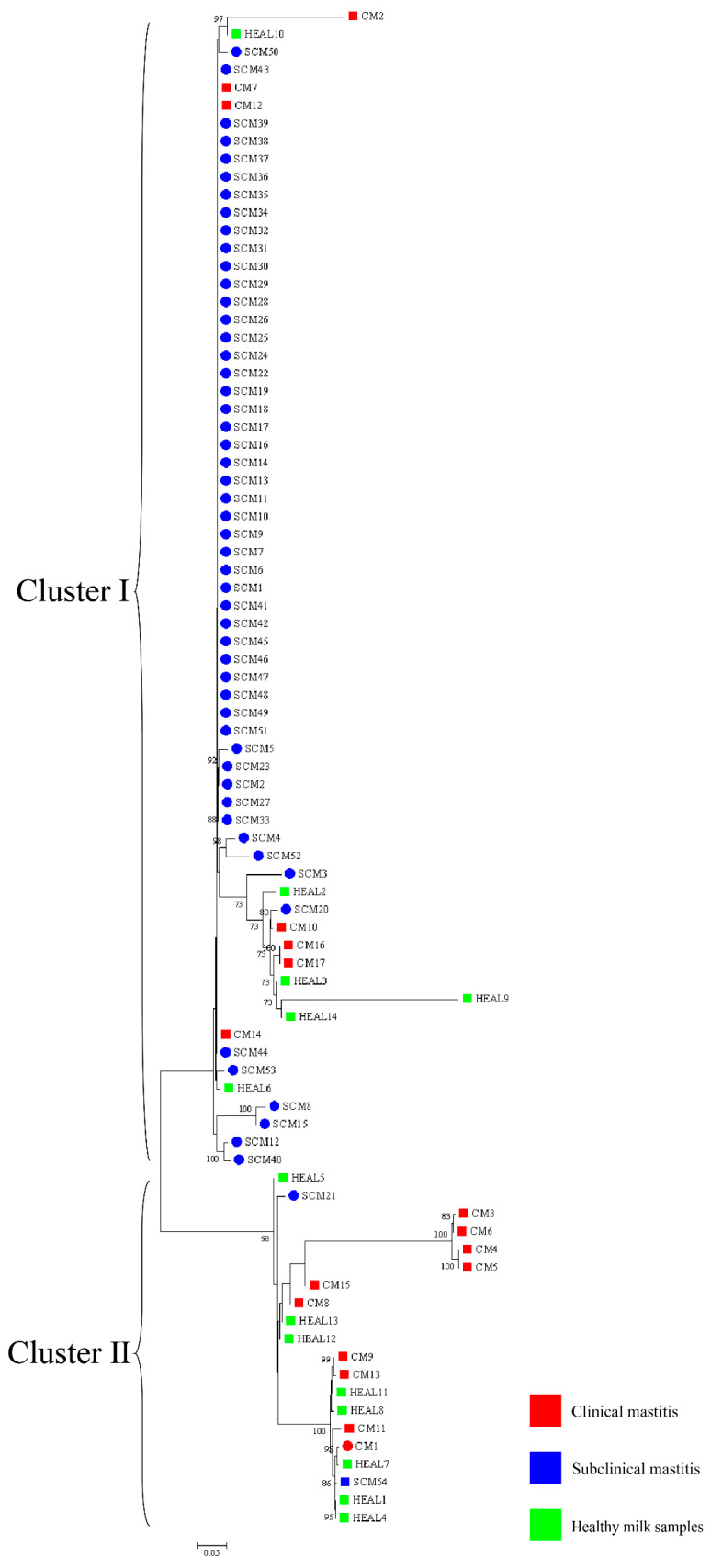
NJ trees based on the *gyrB*-16S rRNA-*secA1*-*hsp65*-*rpoB* gene sequences presenting the phylogenetic relationships of *Nocardia* isolated from clinical mastitis, subclinical mastitis, and healthy milk samples. The scale bars represent 0.05 substitutions per nucleotide position. ○ represents farm A, □ represents farm B.

Phylogenetic analysis was performed based on a combination of five genes (*gyrB*-16S rRNA-*secA1*-*hsp65*-*rpoB*) containing all isolates (Figure 4). Across farms A and B combined, 6 and 8 isolates from HEAL samples were classified into clusters I and II, respectively, yielding an SID of 0.53 (95% CI: 0.46–0.60). Among CM isolates, 7 were assigned to cluster I and 10 to cluster II, with an SID of 0.51 (95% CI: 0.45–0.58). For SCM samples, 58 isolates were grouped into cluster I and 1 into cluster II, resulting in an SID of 0.037 (95% CI: 0.029–0.048). At farm A, 1 isolate from a CM case was classified into cluster II. Among SCM samples, 52 isolates were grouped into cluster I and 1 into cluster II, producing an SID of 0.038 (95% CI: 0.029–0.049). At farm B, 1 SCM isolate was assigned to cluster I. For CM samples, 7 and 9 isolates were classified into clusters I and II, respectively, with an SID of 0.53 (95% CI: 0.46–0.59). Additionally, 6 and 8 HEAL isolates were distributed across clusters I and II, yielding an SID of 0.53 (95% CI: 0.46–0.60).

#### 3.4.3. *gyrb*-*hsp65*-*secA1*-*rpoB*-*rpoA*-*recA*-*trpB*

**Figure 5:**
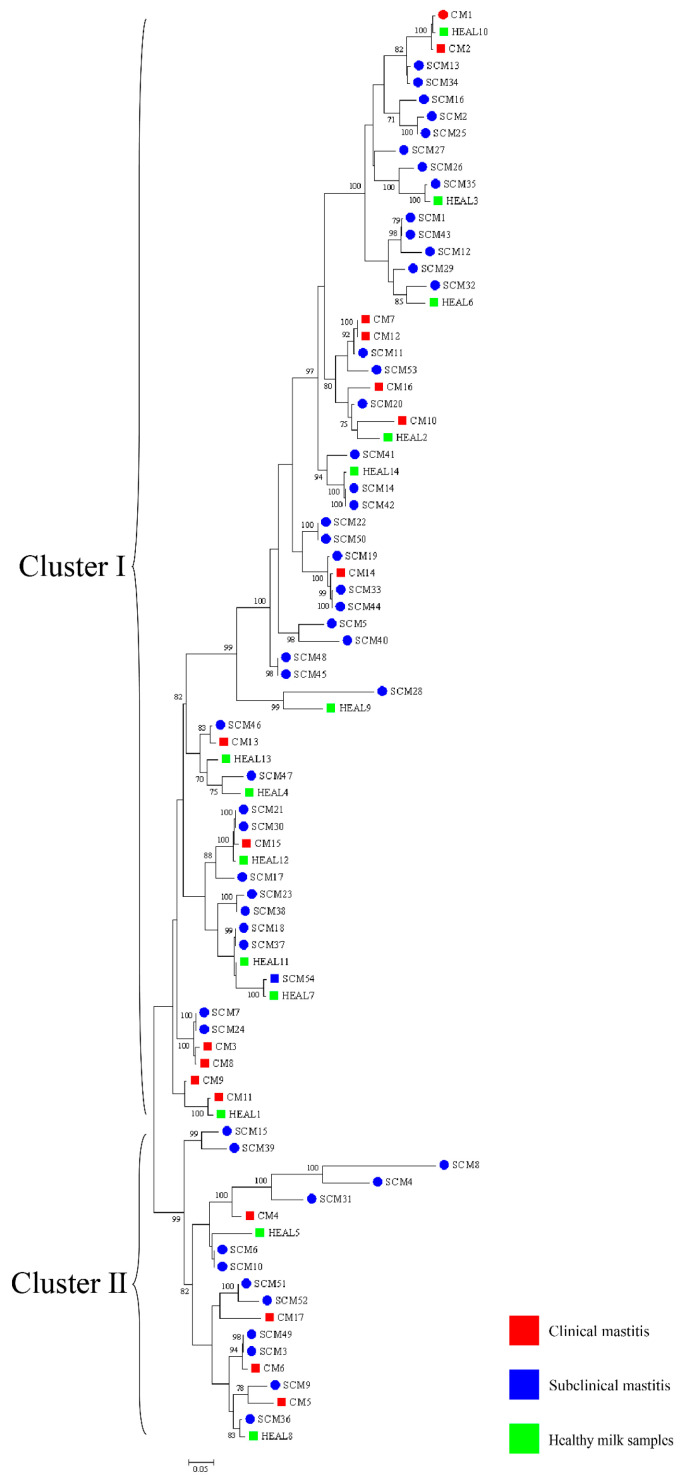
NJ trees based on the *gyrb*-*hsp65*-*secA1*-*rpoB*-*rpoA*-*recA*-*trpB* gene sequences presenting the phylogenetic relationships of *Nocardia* isolated from clinical mastitis, subclinical mastitis, and healthy milk samples. The scale bars represent 0.05 substitutions per nucleotide position. ○ represents farm A, □ represents farm B.

Phylogenetic analysis was performed based on a combination of seven genes (*gyrb*-*hsp65*-*secA1*-*rpoB*-*rpoA*-*recA*-*trpB*) containing all isolates (Figure 5). Across all samples, 13 and 4 CM isolates were classified into clusters I and II, respectively, resulting in a Simpson’s Index of Diversity (SID) of 0.38 (95% CI: 0.30–0.47). Among SCM isolates, 41 were assigned to cluster I and 13 to cluster II, with an SID of 0.37 (95% CI: 0.33–0.42). For HEAL samples, 12 isolates were grouped into cluster I and 2 into cluster II, yielding an SID of 0.26 (95% CI: 0.19–0.36). At farm A, 1 CM isolate was classified into cluster I. Among SCM isolates, 40 were assigned to cluster I and 13 to cluster II, producing an SID of 0.38 (95% CI: 0.33–0.42). At farm B, 1 SCM isolate was classified into cluster I. For CM samples, 12 and 4 isolates were grouped into clusters I and II, respectively, resulting in an SID of 0.40 (95% CI: 0.32–0.49). Among HEAL samples, 12 were assigned to cluster I and 2 to cluster II, with an SID of 0.26 (95% CI: 0.19–0.36).

### 3.5. Prevalence of Nocardia in Environmental Samples

On farm A, a total of 144 environmental samples were tested using TaqMan qPCR, among which 30 (20.8%) tested positive for *N. cyriacigeorgica*. Positive detections were identified in 12 types of sample sources, including milking equipment (ME), hands of the milking personnel (Hmp), milking parlor aisle, cow hoof, freshly prepared clean bedding (Fbf), bedding, feed, teat skin, interdigital spaces (Ids), nasal swabs, saliva, and feces. Among these, nasal samples showed the highest prevalence (75.0%), followed by milking parlor aisle (50.0%) and ME (41.7%), while the lowest prevalence (8.3%) was observed in samples from the Hmp and cow hooves. On farm B, a total of 144 environmental samples were collected and tested using TaqMan qPCR, among which 33 samples (22.9%) tested positive for *N. cyriacigeorgica*. Positive detections were identified in 9 types of sample sources, including ME, milking parlor aisle, Fbf, bedding, feed, teat skin, Ids, nasal swabs, and feces. Among these, teat skin and Ids exhibited the highest prevalence (75.0%), while Fbf, bedding, feed, and feces showed the lowest prevalence, each with a positive rate of 25.0% (Table 6).

To evaluate the association between sample source and *N. cyriacigeorgica* detection, logistic regression analyses were performed for two farms, categorizing samples into cowshed, milking parlor, and cow-related groups. Using cowshed samples as the reference, farm A showed significantly higher odds of detection in the milking parlor (OR = 5.57, 95% CI: 1.64–25.7, *p* = 0.011) and cow-related samples (OR = 6.18, 95% CI: 1.84–28.3, *p* = 0.007). In farm B, the odds were moderately higher for cow-related samples (OR = 4.20, 95% CI: 0.53–5.21, *p* = 0.007), whereas milking parlor samples did not show a statistically significant increase (OR = 1.62, 95% CI: 1.56–12.7, *p* = 0.402) (Table 7). These results consistently suggest that *N. cyriacigeorgica* contamination is more likely to occur on surfaces with direct animal contact and in milking environments than in general cowshed areas, rather than transmission occurring between cows.

## 4. Discussion

*Nocardia cyriacigeorgica*, a pathogenic species within the genus *Nocardia*, was initially classified as type VI of *N. asteroides* [29] and is widely recognized as an opportunistic pathogen in humans, where it causes severe infections. Although our study focuses on bovine mastitis, insights from human nocardiosis highlight its pathogenic potential. In recent years, *N. cyriacigeorgica* has also been isolated from bovine milk samples associated with mastitis worldwide, contributing to significant economic losses in the dairy industry [5,7,8]. During the mastitis outbreak investigated in this study, *N. cyriacigeorgica* was detected at relatively high prevalence rates in milk samples from clinical mastitis cases (CM, 22.7%), subclinical mastitis (SCM, 11.2%), and even apparently healthy cows (HEAL, 28.0%), in agreement with previous reports from China [30]. Notably, the distribution of isolates differed between the two farms: Farm A yielded more isolates, mainly from subclinical mastitis cases, whereas Farm B contributed mostly from clinical mastitis cases with only a single subclinical isolate. This difference may be explained by variations in management practices, diagnostic approaches, or sampling intensity, but could also reflect genuine epidemiological differences and distinct outbreak dynamics at each site. All samples were collected during an active outbreak, offering a unique opportunity to examine the genetic diversity of isolates under conditions of elevated pathogen pressure and environmental exposure. Whole genome sequencing showed closely related *N. cyriacigeorgica* across clinical, subclinical, and healthy milk samples. This low divergence is compatible with exposure to shared farm sources (e.g., equipment, bedding, feed), but it does not identify the source or the direction of spread. Because we did not culture and sequence environmental isolates, we cannot distinguish direct environmental contamination, a single introduction with limited within-farm spread, from repeated reintroductions via external inputs (e.g., feed from the same fields). Given *Nocardia*’s ecology as an environmental opportunist rather than a contagious mastitis pathogen, we view environmental exposure as plausible, not proven, in this dataset.

Accurate identification of *Nocardia* species is essential for epidemiological investigations and clinical management, with full-length 16S rRNA gene sequencing being the gold standard for species-level confirmation [31]. In this study, 16S rRNA sequencing and phylogenetic tree construction revealed a low level of genetic diversity among *N. cyriacigeorgica* strains isolated from clinical mastitis (CM), subclinical mastitis (SCM), and healthy milk samples (HEAL), consistent with previous findings on the genetic relatedness between *N. cyriacigeorgica*, *N. asteroides*, and *N. abscessus* [30]. To enhance phylogenetic resolution, multilocus sequence analysis (MLSA) based on three- and five-gene combinations was also performed. Notably, MLSA results demonstrated a high degree of homogeneity among isolates, particularly those obtained from mastitis cases, suggesting the possible existence of a dominant or clonally derived strain circulating within the farms. Comparable patterns of clonal dissemination have been observed in recent molecular studies of mastitis pathogens. For example, Fu et al. reported extensive genetic diversity and antimicrobial resistance among *Klebsiella pneumoniae* isolates from bovine mastitis using WGS and MLST [13]. Wang et al. further characterized 108 isolates from dairy farms and identified multiple virulence and resistance gene clusters, with indications of local clonal expansion. These findings reinforce the hypothesis that under specific environmental and management conditions, certain strains may dominate through shared reservoirs and transmission pathways [12]. These findings imply that *N. cyriacigeorgica* may spread within farms via environmental routes under specific conditions, driven by shared environmental sources or management practices, rather than by direct transmission between cows. This highlights the need for targeted biosecurity and hygiene interventions to prevent within-farm spread, thereby safeguarding both animal health and farm productivity.

*Nocardia* spp., widely distributed in soil, water sources, and organic matter, demonstrates remarkable ecological adaptability [32]. Its frequent detection in cattle farm environments, particularly in manure, bedding straw, and wastewater, reinforces its classification as an environmental pathogen [33]. In this study, *N. cyriacigeorgica* DNA was detected by qPCR at multiple on-farm sites on both farms, with the highest qPCR-positive frequencies in nasal swabs (75.0%), teat skin and interdigital spaces (75.0%), milking parlor aisles (50.0%), and equipment (41.7–50.0%). Lower qPCR-positive frequencies (8.3–25.0%) were observed in bedding, feed, feces, cow hooves, and personnel hands, while water, manure residue, and soil tested negative by qPCR in our assays. These findings suggest persistent contamination across multiple environmental sites, with repeated animal contact likely contributing to sustained infections. Previous studies have identified recycled manure solids as a major risk factor for environmental mastitis, due to their capacity to retain high bacterial loads and direct contact with the teat end [34,35]. Our results align with these findings, suggesting that recycled manure bedding may serve as a stable reservoir facilitating the persistence of *N. cyriacigeorgica* within dairy herds. A likely cycle involves contaminated feed colonizing the nasal or oral cavities, subsequent fecal shedding, and recycled bedding, leading to re-exposure via teat skin and hooves. This highlights the importance of farm management practices, particularly bedding recycling and hygiene protocols, in sustaining or limiting pathogen dissemination. Comparable to *Klebsiella pneumoniae* in Scottish dairy herds [36], *N. cyriacigeorgica* may establish stable environmental reservoirs with significant epidemiological impact.

**Figure 6:**
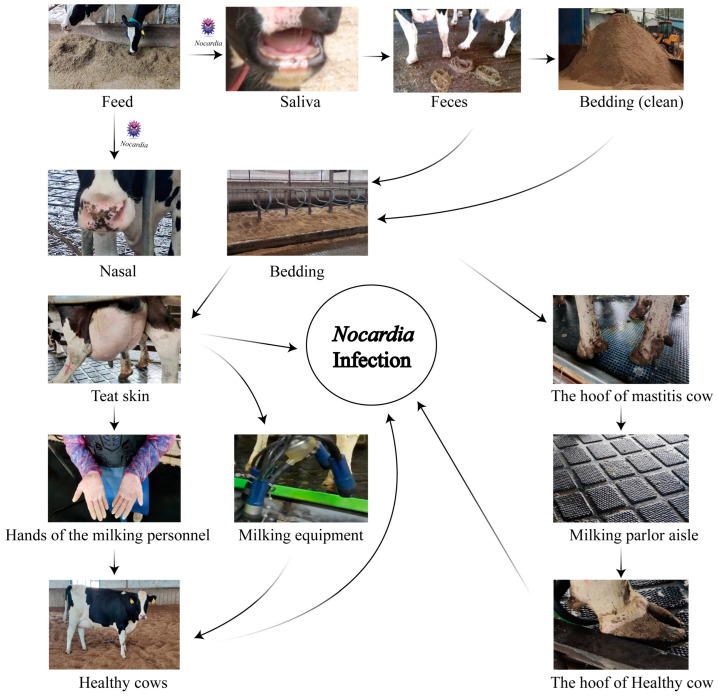
Schematic of the hypothetical transmission of *Nocardia* in dairy farms.

During the milking process, *N. cyriacigeorgica* present on the teat skin may invade the mammary gland, leading to intramammary infections. Contaminated teat skin can also transfer bacteria to the milking equipment liners, posing a risk of subsequent infection in healthy cows. Similarly, bacteria on the hooves may be deposited onto rubber mats in the milking parlor, increasing the risk of environmental transmission and hoof-related infections (Figure 6). Notably, nocardial mastitis was predominantly diagnosed in clinical cases within dairy herds characterized by poor environmental hygiene between milking sessions and inappropriate intramammary therapy practices [37]. Furthermore, phylogenetic analyses based on three-gene (16S rRNA–*gyrB*–*hsp65*), five-gene (*gyrB*–16S rRNA–*secA1*–*hsp65*–*rpoB*), and seven-gene (*gyrB*–*hsp65*–*secA1*–*rpoB*–*rpoA*–*recA*–*trpB*) MLSA schemes revealed that isolates from farms A and B clustered separately, indicating genetic heterogeneity between farms. In contrast, isolates within each farm exhibited high genetic similarity over a short time frame, suggesting environmental clonal persistence, where specific lineages persist in farm environments and intermittently infect cows. Notably, the clustering results also varied among the three-gene, five-gene, and seven-gene MLSA schemes, reflecting the locus-dependent resolution of multilocus analyses. The three-gene set includes highly conserved loci, particularly 16S rRNA, which limits discriminatory power. The five-gene scheme improves resolution by incorporating additional housekeeping genes, while the seven-gene scheme captures even greater variation due to the inclusion of faster-evolving loci. In addition, locus-specific effects of recombination and horizontal gene transfer may contribute to conflicting topologies. These findings emphasize that MLSA outcomes depend strongly on gene selection, and whole genome sequencing provides the most robust framework for high-resolution phylogenetic inference. Previous studies have shown that the burden of human nocardiosis varies across geographic regions [11]. Consistent with this, our findings suggest that the genetic diversity and persistence of *N. cyriacigeorgica* are influenced by geographic location and farm-specific environmental conditions.

Currently, the antibiotic treatment of *N. cyriacigeorgica* infections remains challenging. Therapy often requires prolonged courses, yet the clinical outcomes are frequently suboptimal, potentially resulting in chronic inflammation of the mammary gland, reduced milk yield, and compromised overall health of affected cows [38,39]. In this study, antimicrobial susceptibility testing (AST) showed universal resistance to clarithromycin and rifampin and high resistance to ciprofloxacin, confirming the limited utility of these agents. In contrast, all isolates were susceptible to amikacin and tobramycin, suggesting aminoglycosides as the most reliable therapeutic option. Moderate resistance to doxycycline and moxifloxacin highlights variable efficacy and reinforces the need for susceptibility-guided therapy. These patterns accord with Brazilian reports [37,40] where environmental lapses have been implicated and multidrug-resistant *Nocardia* have emerged, underscoring the need for environmental control and cautious antimicrobial stewardship. To prevent further spread, affected cows should be promptly isolated or removed, while future research should integrate AST profiling with studies on virulence and host–pathogen interactions to inform more effective control strategies.

In this study, we investigated the genetic diversity of *N. cyriacigeorgica* within two dairy herds under outbreak conditions. The close clustering of milk isolates on whole genome sequencing and multilocus sequence analysis, together with qPCR detection at shared contact sites, is consistent with indirect exposure involving milking parlor aisles, shared equipment, and high animal density. However, environmental samples were not cultured and environmental isolates were not sequenced, so the data do not allow attribution of source or inference about transmission direction. In the absence of established interpretive standards for bovine nocardial mastitis, we used homology-based comparisons to describe relatedness among clinical, subclinical, and healthy milk isolates, and these comparisons require further methodological validation before they can inform attribution. The findings generate hypotheses regarding environmental exposure pathways, but repeated acquisition from environmental reservoirs cannot be concluded from the present dataset. Alternative explanations remain plausible, including a single introduction followed by limited within-farm spread or repeated reintroductions through external inputs such as feed from the same fields. From a practical standpoint, the results support prioritizing control at shared exposure points, including teat-skin hygiene, sanitation of liners and other equipment, management of bedding, and secure storage of feed, while confirmation should rely on longitudinal environmental culture with parallel sequencing of environmental and milk isolates. This investigation was conducted on two farms in Hebei and Shandong over a short interval; therefore, the findings should be considered preliminary and context specific, and multi-farm longitudinal studies with environmental culture and sequencing are warranted.

## 5. Conclusions

This study examined *Nocardia cyriacigeorgica* in two Chinese dairy herds during mastitis outbreaks. The pathogen was isolated from clinical, subclinical, and healthy milk, and qPCR detected its DNA at multiple on-farm sites. Whole genome and multilocus sequence analyses showed low diversity among milk isolates, with most belonging to closely related lineages. These patterns are consistent with exposure through shared environmental contact points within farms; the specific source remains undetermined. The findings support prioritizing hygiene and biosecurity at shared contact points, and multi-farm longitudinal studies that include environmental culture and sequencing are needed to validate and extend these observations.

## Figures and Tables

**Table 1:** Characteristics of the 2 Chinese dairy farms with outbreaks of *Nocardia* mastitis.

Source	Farm A	Farm B
Herd size (lactating cows)	4770	11,092
Average bulk tank SCC (cells/mL)	153,930	131,900
Bedding material	Recycled manure solids (50–60% DM)	Recycled manure solids (50–60% DM)
Frequency of cleaning stalls (times/d)	1	1
Frequency of replacing bedding (times/wk)	3	3
Alley scraping (passes/h)	1	1
Milking parlor	Parallel (double 32)	Rotary (80 sites)
Frequency of milking (times/d)	3	3
Teat disinfectants	0.25% and 0.5% iodine solution for pre- and post-milking	0.25% and 0.5% iodine solution for pre- and post-milking

**Table 2:** Sampling methods for extramammary and environmental samples on farms A and B.

Source	Type	Sterile Swab	Sterile Ziplock Bag	Disposable Straw	PBS
Milking parlor	Me ^1^	1			5 mL
	Hmp ^2^	1			5 mL
	Milking parlor aisle	1			5 mL
	Cow hoof	1			5 mL
Cowshed	Fbf ^3^		50 mg		20 mL
	Rsc ^4^		50 mg		20 mL
	Bedding		50 mg		20 mL
	Sbb ^5^		50 mg		20 mL
	Feed		50 mg		20 mL
	Water			5 mL	
Cows	Teat skin	1			5 mL
	Teat orifice	1			5 mL
	Ids ^6^	1			5 mL
	Nasal	1			5 mL
	Saliva	1			5 mL
	Feces	1			5 mL

^1^ Me = Milking equipment; ^2^ Hmp = Hands of the milking personnel; ^3^ Fbf = Freshly prepared clean bedding in the dairy farm; ^4^ Rsc = Residue left after wet and dry separation of cow manure; ^5^ Sbb = Soil under the bedding of the perinatal barn; ^6^ Ids = Interdigital spaces.

**Table 3:** Primer and probe sequences.

Serial Number	Primer Name	Primer Sequence (5′→3′)	Fragment Size (bp)	Product Size (bp)
1	N16S-F	CTGGGATAAGCCTGGGAAAC	20	128
2	N16S-R	TAGGCCATTACCCCACCAAC	20	
3	N16S-P	ACCTTACATCGCATGGTGTTTGGTGGA	27	

**Table 4:** Isolation of *Nocardia cyriacigeorgica* from the 2 dairy farms.

Source	Farm A		Farm B		Total	
	No. Samples	*N. cyriacigeorgica* (No. [%])	No. Samples	*No. cyriacigeorgica* (No. [%])	No. Samples	*N. cyriacigeorgica* (No. [%])
CM	45 ^b^	1 (2.2) ^b^	30 ^b^	16 (53.3) ^a^	75 ^b^	17 (22.7) ^a^
SCM	431 ^a^	53 (12.3) ^a^	50 ^a^	1 (2.0) ^c^	481 ^a^	54 (11.2) ^b^
BTM	30 ^c^	0 (0.0) ^b^	30 ^b^	0 (0.0) ^c^	60 ^c^	0 (0.0) ^d^
HEAL	0 ^d^	0 (0.0) ^b^	50 ^a^	14 (28.0) ^b^	50 ^d^	14 (28.0) ^c^
Total	506	54 (10.7)	160	31 (19.4)	666	85 (12.8)

^a,b,c,d^ Within a row, proportions without a common letter indicated a difference (*p* < 0.05) between the 2 farms. CM: clinical mastitis, SCM: subclinical mastitis, BTM: bulk tank milk, HEAL: healthy milk samples.

**Table 5:** Antibiotic susceptibility results for 85 *N. cyriacigeorgica* isolates.

Drug ^1^	Distribution of MIC (μg/mL)											CLSI Break Points ^2^			Resistance Rate (%)
	0.06	0.12	0.25	0.5	1	2	4	8	16	32	>32	R	I	S	
CLR	0	0	0	0	0	0	0	0	6	27	52	≥8	4	≤2	100
RIF	0	0	0	0	0	0	0	23	27	9	26	≥4	2	≤1	100
MXF	0	1	2	3	6	49	23	1	0	0	0	≥4	2	≤1	28.2
CIP	0	1	1	0	2	2	9	43	23	4	0	≥4	2	≤1	92.9
DOX	0	0	0	0	0	6	52	8	11	8	0	≥8	2–4	≤1	31.8
AMK	0	0	0	19	38	22	4	2	0	0	0	≥16		≤8	0
TOB	0	0	0	2	51	24	8	0	0	0	0	≥16	8	≤4	0

^1^ CLR = clarithromycin; RIF = rifampin; MXF = moxifloxacin; CIP = ciprofloxacin; DOX = doxycycline; AMK = amikacin; TOB = tobramycin. ^2^ R = resistant; I = intermediate; S = susceptible.

**Table 6:** Distribution of *Nocardia* on farms A and B, according to origin of sample.

Source	Type	Farm A			Farm B		
		No. Samples	*N. cyriacigeorgica* (No. [%])	Mean Ct	No. Samples	*N. cyriacigeorgica* (No. [%])	Mean Ct
Milking parlor	Me ^1^	12	5 (41.7)	28.95 ± 0.72	12	6 (50.0)	27.41 ± 0.93
	Hmp ^2^	12	1 (8.3)	31.9	12	0 (0.0)	
	Milking parlor aisle	12	6 (50.0)	29.03 ± 1.12	12	3 (25.0)	28.1 ± 0.55
	Cow hoof	12	1 (8.3)	26.98	12	0 (0.0)	
Cowshed	Fbf ^3^	8	1 (12.5)	13.44	8	2 (25.0)	15.65 ± 0.59
	Rsc ^4^	8	0 (0.0)		8	0 (0.0)	
	Bedding	8	1 (12.5)	21.77	8	2 (25.0)	21.85 ± 0.06
	Sbb ^5^	8	0 (0.0)		8	0 (0.0)	
	Feed	8	1 (12.5)	27.93	8	2 (25.0)	28.9 ± 0.64
	Water	8	0 (0.0)		8	0 (0.0)	
Cows	Teat skin	8	3 (37.5)	24.38 ± 0.47	8	6 (75.0)	21.1 ± 1.34
	Teat orifice	8	0 (0.0)		8	0 (0.0)	
	Ids ^6^	8	3 (37.5)	19.91 ± 0.71	8	6 (75.0)	26.98 ± 1.27
	Nasal	8	6 (75.0)	17.73 ± 1.06	8	4 (50.0)	27.93 ± 0.82
	Saliva	8	1 (12.5)	22.57	8	0 (0.0)	
	Feces	8	1 (12.5)	28.38	8	2 (25.0)	30.2 ± 0.22

^1^ Me = Milking equipment; ^2^ Hmp = Hands of the milking personnel; ^3^ Fbf = Freshly prepared clean bedding in the dairy farm; ^4^ Rsc = Residue left after wet and dry separation of cow manure; ^5^ Sbb = Soil under the bedding of the perinatal barn; ^6^ Ids = Interdigital spaces.

**Table 7:** Comparison of *Nocardia* detection risk across sample sources in farms A and B (reference: cowshed).

Sample Source (Vs Cowshed)	Farm A			Farm B		
	Odds Ratio (OR)	95% CI	*p*-Value	Odds Ratio (OR)	95% CI	*p*-Value
Cowshed (Ref)	1.0	–	–	1.0	–	–
Milking parlor	5.57	1.64–25.7	0.011	1.62	0.53–5.21	0.402
Cows	6.18	1.84–28.3	0.007	4.20	1.56–12.7	0.007

## Data Availability

The original contributions presented in this study are included in the article. The raw sequencing data have been deposited in the NCBI BioProject database under accession number PRJNA1300593. Further inquiries can be directed to the corresponding authors.
